# A Case of Bullous Skin Disease Presenting with Odynophagia: A Diagnostic Challenge

**DOI:** 10.1155/2016/2839104

**Published:** 2016-03-14

**Authors:** G. Kravvas, D. Veitch, C. M. Perrett

**Affiliations:** Department of Dermatology, University College London Hospitals, London NW1 2BU, UK

## Abstract

We report a case of Epidermolysis Bullosa Acquisita (EBA) that presented as a diagnostic challenge. A 60-year-old Qatari lady presented with odynophagia, oral ulceration, and weight loss. Multiple physicians investigated her for over 6 months with a multitude of tests and serial gastroscopies, all of which failed to reach a conclusive diagnosis. Only after referral to a dermatologist and full body examination was diagnosis finally achieved. After reviewing the literature, we provide a summary of EBA and highlight the importance of comprehensive clinical reviews in order to avoid unnecessary morbidity.

## 1. Introduction

Epidermolysis Bullosa Acquisita (EBA) is a rare blistering disease that can affect many sites and present with various symptoms. It is a well-recognised entity within the field of dermatology and can be managed well with immunosuppression.

This case is notable because the patient was investigated and treated by nondermatologist physicians who failed to perform a comprehensive skin examination. As a consequence, she suffered unnecessary, significant morbidity over a long period of time. Once she was referred appropriately and the necessary steps were undertaken, diagnosis became quickly apparent and the appropriate treatment was initiated successfully.

## 2. Case Report

We report the case of a 60-year-old Qatari lady who was referred with a history of weight loss, odynophagia, and oral aphthous ulceration.

Over a period of 6 months, she was thoroughly investigated by the local medical team with blood tests and a full body CT scan, all of which were unremarkable. She underwent serial gastroscopies (OGDs) revealing superficial ulceration of the oropharynx and proximal oesophagus, with biopsy results suggestive of herpes simplex virus-related pathology. Virology however was negative.

A prolonged course of high-dose valacyclovir failed to provide any benefit.

**Figure 1:**
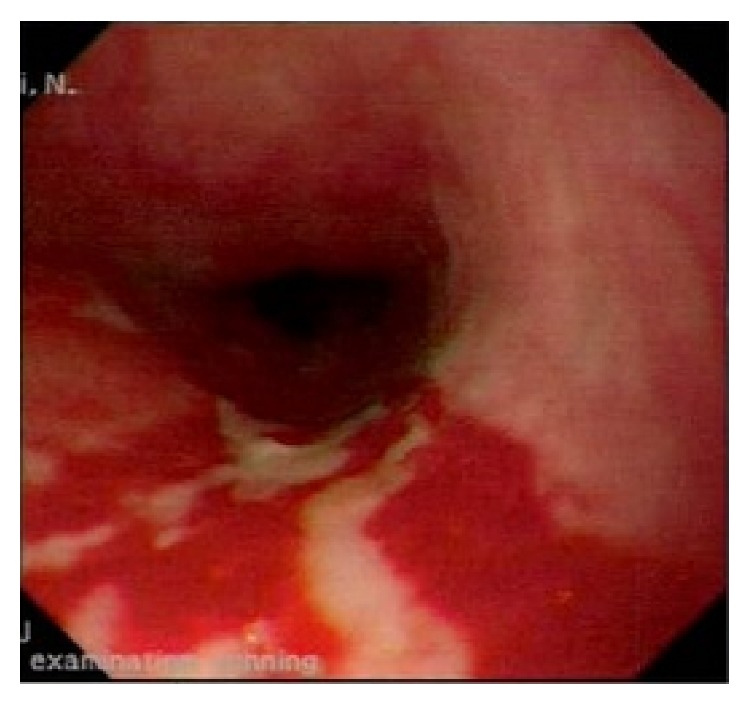
Midoesophageal endoscopic image showing superficial ulceration.

She continued to deteriorate and was referred to a gastroenterology clinic where alternative diagnoses were considered. Further endoscopies were undertaken which confirmed extensive superficial ulceration from the oropharynx to the distal oesophagus (Figure 1). Histology was not typical of herpes and virology was negative.

Subsequently, she was referred to a dermatologist for investigation of oral ulceration. Further examination revealed an erythematous vesiculobullous lesion on her left arm with widespread patchy postinflammatory hyper- and hypopigmentation on both arms. Further direct questioning elicited a 6-month history of intermittent, itchy skin lesions all of which resolved spontaneously leaving residual pigment irregularity. Examination of the buccal mucosa revealed extensive ulceration.

Examination of the hair, scalp, nails, genitalia, and eyes was unremarkable.

**Figure 2:**
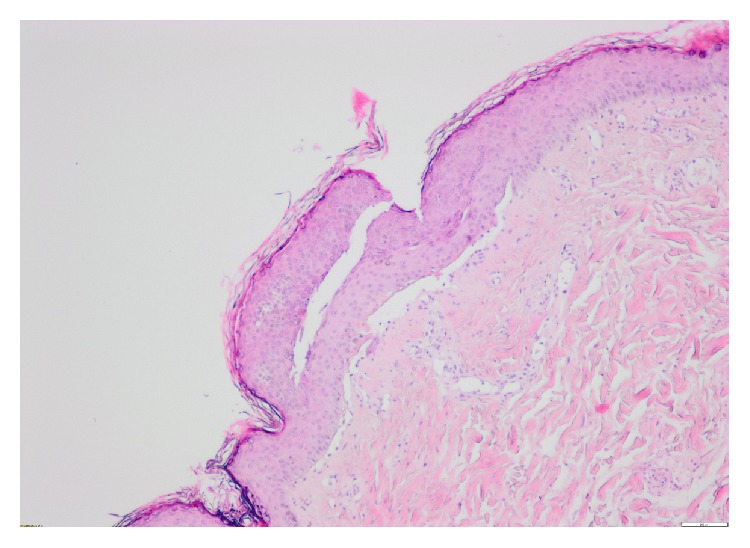
Biopsy of blister showing neutrophils within the blister cavity (haematoxylin and eosin, original magnification ×40).

**Figure 3:**
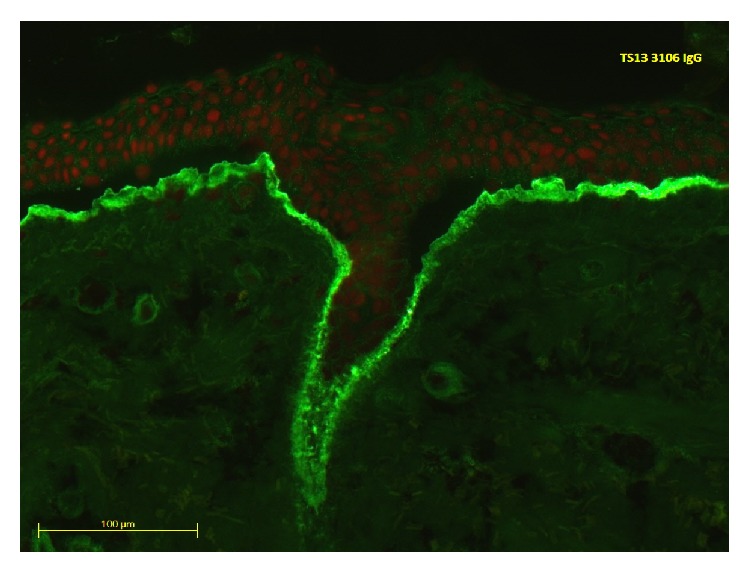
Direct IMF displaying linear deposition of IgG, IgA, and C3 at the basement membrane zone.

Skin biopsy displayed subepidermal blistering with neutrophils in the blister cavity (Figure 2). Direct immunofluorescence (IMF) showed linear deposition of IgG, IgA, and C3 at the basement membrane zone (BMZ) with immunoreactants localising to the base where the split occurred (Figure 3). Indirect IMF detected IgG anti-BMZ antibodies (titre 1 : 100) and EBA antigen ELISA was positive for anti-collagen 7 antibodies, supporting a diagnosis of Epidermolysis Bullosa Acquisita (EBA).

Treatment with antivirals was stopped and the patient was commenced on treatment with prednisolone 60 mg once daily and Betnesol mouthwash. Although her symptoms improved to an extent, she continued to develop further bullous skin lesions. She was started on treatment with mycophenolate mofetil and was weaned off steroids.

1 year later, her condition is in complete remission with no further odynophagia and no evidence of any skin or mouth lesions.

## 3. Discussion

EBA is a rare blistering disease which, unlike Epidermolysis Bullosa (EB), is acquired, normally presenting in the fourth or fifth decade of life, with a prevalence of 0.2/million people [1]. Type 7 collagen structures are a major component of anchoring fibrils attaching epidermis to dermis. IgG autoantibodies to this leads to the subepidermal bullous disease of EBA [2].

5 distinct phenotypes of EBA have been described [3]: “classical” noninflammatory EBA is the most common form resulting in trauma-induced blistering mainly on extensor surfaces of extremities causing significant scarring and milia on rupture [4]. Inflammatory EBA is subdivided into 4 forms with the predominant form being Bullous Pemphigoid-like presenting with widespread, tense bullae on trauma-prone sites which heal with minimal scarring [5]. Other forms include Brunsting-Perry Pemphigoid-like, linear IgA bullous dermatosis-like and, as in this case report, Cicatricial Pemphigoid- (CP-) like presentation [5].

This case presented with oropharyngeal and proximal oesophageal superficial ulceration and a right forearm pruritic vesicular lesion. Interestingly, the CP form can involve conjunctivae, tracheal, anal, and vaginal ulceration which was not present in this case [6, 7]. Additionally, scarring is normally seen in the CP form; however, this patient had mucosal involvement without scarring which is rarely described [8].

EBA is considered as an autoimmune disorder and so immunosuppression is the mainstay of treatment with prognosis excellent if remission is achieved. Due to its preferable safety profile compared to azathioprine, mycophenolate mofetil is recommended as first line treatment [9].

## 4. Conclusion

This report highlights the importance of full skin and mucosal examination in all patients with odynophagia. When this is not performed in a timely fashion, it can lead to misdiagnosis, unnecessary investigations, and increased morbidity of an otherwise treatable condition.
